# The relationship between public health expenditure and urban economic resilience

**DOI:** 10.3389/fpubh.2025.1550528

**Published:** 2025-02-14

**Authors:** Erdong Chen, Huaxin Zhang

**Affiliations:** ^1^School of Economics, Liaoning University, Shenyang, China; ^2^Faculty of History, Culture, and Tourism, Guangxi Minzu Normal University, Chongzuo, China; ^3^School of Public Administration, South China Agricultural University, Guangzhou, China

**Keywords:** public health expenditure, urban economic resilience, technological innovation, per capita GDP, regional heterogeneity, spatial spillover effects

## Abstract

Achieving urban economic resilience is a critical objective for sustainable development in the face of external shocks. Public health expenditure plays a pivotal role in enhancing urban economic resilience by improving health outcomes, optimizing resource allocation, and strengthening economic capacity to withstand risks. However, the mechanisms through which public health expenditure influences resilience, as well as its regional variations, remain underexplored. This study utilizes panel data from 284 cities in China spanning from 2008 to 2021, constructing an econometric model that incorporates mediating variables such as technological innovation and per capita GDP, to assess both the direct and indirect effects of public health expenditure on urban economic resilience. Additionally, spatial econometric models are employed to further analyze the spatial spillover effects of public health expenditure. The findings reveal that public health expenditure significantly enhances urban economic resilience, with technological innovation and per capita GDP serving as key mediating pathways. Regional analysis shows that the impact is most pronounced in eastern cities, followed by central cities, while the effect in western cities is weaker and, in some cases, negative. Spatial analysis further indicates that public health expenditure has a significant positive spillover effect on neighboring cities, primarily through resource sharing and technology diffusion. This study suggests that optimizing the structure of public health expenditure, increasing infrastructure investment, supporting non-capital and resource-dependent cities, and promoting digital healthcare and regional cooperation are essential to enhancing economic resilience, fostering high-quality urban development, and advancing regional equity.

## 1 Introduction

The interaction between public health issues and economic development has garnered increasing attention in the context of accelerating globalization and urbanization. In recent years, recurrent public health crises have posed profound challenges to global health systems while disrupting the stability and sustainability of regional and global economies. Public health expenditure, as a cornerstone of improving population health and promoting social welfare, plays a vital role in addressing these challenges. Encompassing a wide range of activities—including the development of medical infrastructure, vaccine research and distribution, and disease prevention—public health expenditure directly strengthens the foundational health conditions necessary for economic stability. By optimizing resource allocation and promoting health equity, it bolsters both societal resilience and economic growth (1).

Cities, as the primary hubs of economic activity, face mounting vulnerabilities to external shocks such as natural disasters, economic fluctuations, and public health emergencies. Urban economic resilience—the capacity of cities to withstand, adapt to, and recover from such disruptions—has become a key indicator of their risk management capabilities and long-term viability. Existing research highlights how economic resilience enables cities to adjust their economic structures, optimize resource allocation, and sustain growth amid crises (2). Enhancing urban economic resilience, therefore, is not only critical for a city’s ability to survive and recover during crises but also for achieving high-quality, sustainable development and social stability.

There exists a profound and intrinsic relationship between public health expenditure and urban economic resilience. Public health spending plays a pivotal role in safeguarding the health of urban populations, enhancing resilience to risks, promoting economic structural optimization, and advancing social stability and health equity. As such, it is not only a critical component in cities’ responses to public health crises, but also a key lever in strengthening urban economic resilience. This study addresses three core questions: (1) how public health expenditure affects urban economic resilience; (2) the mechanisms through which this influence operates; and (3) the regional and spatial characteristics of these effects. By providing both theoretical insights and empirical evidence, this work aims to contribute to the growing body of literature on urban economic resilience and to inform policymakers in optimizing public health and economic strategies.

## 2 Literature review

Public health expenditure is a fundamental component of socio-economic development in modern nations. With the increasing frequency of global public health crises, the economic benefits and social impacts of such expenditures have garnered significant attention (3, 4). Economically, public health investment enhances labor productivity by improving population health (5, 6). A healthier workforce demonstrates higher participation rates and greater efficiency, thereby contributing robustly to economic growth (7). Moreover, health investment reduces the burden of medical expenses, freeing up disposable income for businesses and households, which in turn stimulates consumption and investment, driving further economic development (8). Public health expenditure also indirectly fosters per capita GDP growth by improving overall health conditions (9, 10). Empirical evidence highlights a strong positive correlation between per capita GDP growth and public health spending (11). Additionally, the growing demand for health services promotes the expansion of the service sector, optimizes urban industrial structures, and enhances economic diversity and resilience to external shocks (12, 13). From a social perspective, the allocation of public health expenditure is closely tied to achieving social equity (14, 15). Equitable distribution of healthcare resources can narrow health disparities, improve access to medical services for vulnerable populations, and enhance overall societal well-being (16). For developing countries, increasing public health investments in low-income regions is particularly important, as it optimizes resource allocation and fosters balanced regional development (17). To address public health challenges more effectively, many countries are increasing the proportion of their budgets dedicated to public health, with particular emphasis on vaccine development, healthcare infrastructure, and disease prevention (18). Simultaneously, ensuring the equitable distribution of resources—particularly by prioritizing underserved groups and underdeveloped areas—is essential to achieving universal healthcare access and equity (19).

Against the backdrop of globalization and rapid urbanization, enhancing urban economic resilience has emerged as a key concern for scholars and policymakers alike (20–22). Existing studies on economic resilience often focus on dimensions such as technological resilience, resilience measurement, policy orientation, and industrial diversification. Urban economic resilience is typically defined as a city’s ability to withstand, adapt to, and recover from external shocks, encompassing four dimensions: resistance, recovery, regeneration, and adaptability (23). This concept emphasizes not only short-term recovery but also long-term structural adjustments and innovation capacity (24). In terms of technological resilience, research has shown that cities can effectively respond to and adapt to economic shocks by optimizing technological structures and enhancing technological capabilities, underscoring the critical role of technological resilience in urban economic development (25, 26). Economic resilience is commonly measured using either composite indicator systems or single-indicator methods. For instance, some scholars have developed integrated ecological risk assessment frameworks to demonstrate the long-term Granger causality between land-use changes and ecological risks (27). Other studies compare economic resilience across urban and rural regions relative to regional averages, calculating the economic resilience of NUTS-3 regions (28). Additionally, by analyzing actual wage levels against counterfactual predictions, researchers have assessed urban performance during economic shocks (29). Spatial panel data models have also been employed to forecast employment growth rates, providing insights into the resilience of different regions when confronted with economic disruptions (30). The determinants of economic resilience are multi-dimensional and multi-layered. A diversified economic structure effectively mitigates risks by reducing the impact of external shocks on specific industries or economies. Regions with higher levels of education and skilled labor markets adapt more rapidly to economic changes, fostering innovation and growth. Moreover, robust innovation ecosystems create new growth drivers through technological advancements, knowledge diffusion, and business incubation. Historical and cultural traditions also influence economic resilience by shaping institutional quality, social behavior, and economic structures (31, 32). Scholars argue that optimizing industrial structures is a vital strategy for enhancing economic resilience. Strengthening innovation capacity enables regions to quickly adjust and achieve sustainable growth following crises. Additionally, policies such as government subsidies and tax incentives help businesses and households weather economic hardships. Integration into global value chains also provides regions with access to critical resources and opportunities (33, 34).

In summary, most existing studies either focus on public health expenditure or analyze economic resilience, but few comprehensively examine the interplay between these two dimensions (35, 36). Furthermore, the majority of research linking public health expenditure and economic development emphasizes economic growth, with limited attention paid to urban economic resilience. This gap underscores the need for empirical analysis at the regional level. Building on prior research, this study explores the relationship between public health expenditure and urban economic resilience using data from 284 Chinese cities at the prefecture level and above between 2008 and 2021. Through both theoretical and empirical approaches, it examines the impact of public health expenditure on urban economic resilience. The marginal contributions of this paper are as follows: (1) it investigates urban economic resilience from the perspective of public health expenditure, examining the relationship between the two and addressing gaps in existing research; (2) it provides a theoretical analysis of the mechanisms through which public health expenditure influences urban economic resilience via technological innovation and per capita GDP, uncovering the underlying link between public health spending and economic resilience; (3) it validates the reliability and applicability of the findings through various robustness checks, including excluding data from municipalities directly under the central government, altering the dependent variables, and adjusting estimation methods; (4) it analyzes the significant regional disparities in public health expenditure across eastern, central, and western cities and explores the potential causes, offering new theoretical insights for regional policy development; and (5) it employs a two-way fixed-effects spatial Durbin model to examine the spatial impact of public health expenditure on urban economic resilience and conducts a spillover effects analysis, aiming to provide recommendations for fostering high-quality regional economic development.

## 3 Theoretical foundations and research hypotheses

### 3.1 Direct impact of public health expenditure on urban economic resilience

Public health expenditure directly enhances urban economic resilience by improving public health, upgrading labor quality, and strengthening the economy’s ability to withstand risks. By improving healthcare conditions, reducing the spread of diseases, and increasing labor productivity, public health spending mitigates health-related risks and social burdens that impact economic operations. Furthermore, it drives the growth of healthcare-related industries, creating employment opportunities and injecting new vitality into local economies (37). Equitable distribution of public health resources helps narrow regional disparities in access to medical care, promoting balanced allocation of economic resources and improving overall economic resilience. By advancing healthcare infrastructure and emergency management systems, public health spending significantly strengthens urban preparedness for crises. In the event of natural disasters or pandemics, cities can recover economic activity more quickly, shortening the recovery period. More importantly, increased public health spending fosters confidence in the future among residents, encouraging consumption and investment, and ultimately creating a favorable social environment for economic growth (38).

### 3.2 Indirect impact of public health expenditure on urban economic resilience

Public health expenditure indirectly enhances urban economic resilience through technological innovation. First, it provides critical financial support for research and development in the medical sector, driving advancements in innovative drugs, medical devices, and diagnostic technologies. These innovations improve the efficiency of disease prevention, diagnosis, and treatment, reducing public health risks and bolstering the economy’s capacity to withstand shocks (39). Second, public health expenditure facilitates the upgrading of the medical technology industry by integrating advanced technologies such as biotechnology, artificial intelligence, and big data. These advancements inject new momentum into the economy, enhance the efficiency and coverage of public health services, and improve the adaptive and regenerative capacity of urban economies. Additionally, technological innovation in public health significantly improves cities’ emergency management capabilities. Innovations such as vaccine development, rapid diagnostic tools, and big data monitoring systems enable governments to respond swiftly to public health crises, minimizing disruptions to economic activity (40).

Public health expenditure also strengthens urban economic resilience by boosting per capita GDP. Improved healthcare infrastructure and higher-quality services contribute to better public health outcomes, increasing labor productivity and efficiency. A healthier workforce reduces the disruptive effects of illness on economic activities, raising labor force participation and driving economic growth. These factors support the growth of per capita GDP, providing cities with a more stable economic foundation and enhancing their capacity to resist and recover from external shocks (41). Moreover, public health expenditure optimizes social resource allocation and reduces health inequalities, ensuring that more people have equitable access to medical services. This improves overall social welfare, increases residents’ willingness to consume and invest, and drives income growth and economic development, creating greater economic buffers (42). As public health and income levels rise, demand for high-quality services also increases, spurring the development of high-value-added industries such as healthcare and service sectors. This diversification broadens economic growth channels, enhances economic flexibility, and further consolidates urban economic resilience (43).

## 4 Model, data, and methods

### 4.1 Model design

This study adopts a mediation effect model to analyze both the direct impact of public health expenditure on urban economic resilience and its indirect effects through mediating variables. The objective is to comprehensively reveal the mechanisms through which public health expenditure influences urban economic resilience.

Mediation effect analysis introduces mediating variables to clarify the intrinsic relationship between the primary explanatory variable and the dependent variable. This study identifies two key pathways: Public health expenditure promotes urban economic resilience through technological innovation. Public health expenditure indirectly enhances urban economic resilience by influencing per capita GDP. From an empirical perspective, if public health expenditure-induced changes in technological innovation and per capita GDP significantly improve urban economic resilience, these variables can be regarded as mediators. The two primary methods for mediation effect analysis are:

Stepwise Regression Method: This approach calculates the contribution of each independent variable to the dependent variable, constructing a regression model with strong explanatory power while maintaining low complexity.

Coefficient Product Method: This includes tests such as the Sobel test and the Bootstrap test. Compared to stepwise regression, the Sobel test is a widely used significance testing method for determining whether a mediating variable significantly links the independent and dependent variables.

Based on these theoretical foundations, this study first applies the stepwise regression method for preliminary validation and then employs the Sobel test to ensure the robustness of the results. Accordingly, a baseline regression model (Equation 1) and a mediation effect model (Equations 2, 3) are constructed to conduct the analysis and validation.


(1) $$res_{\mathrm{it}}=\beta_{0}+\beta_{1}pub_{\mathrm{it}}+\underset{j}{\sum }\beta_{j}\times \mathrm{control}+\mu_{t}+\delta_{i}+\varepsilon_{\mathrm{it}}$$

(2) $$M_{\mathrm{it}}=\alpha_{0}+\alpha_{1}pub_{\mathrm{it}}+\underset{j}{\sum }\alpha_{j}\times \mathrm{control}+\mu_{t}+\delta_{i}+\varepsilon_{\mathrm{it}}$$

(3) $${res}_{\mathrm{it}}=\gamma_{0}+\gamma_{1}{\mathrm{pub}}_{\mathrm{it}}+\gamma_{2}M_{\mathrm{it}}+\underset{j}{\sum }\gamma_{j}\times \mathrm{control}+\mu_{t}+\delta_{i}+\varepsilon_{\mathrm{it}}$$

Among the variables, ${res}_{\mathrm{it}}$ represents urban economic resilience, ${\mathrm{pub}}_{\mathrm{it}}$ denotes public health expenditure, and $M_{\mathrm{it}}$ refers to the mediating variables, which in this study include technological innovation and per capita GDP. The index i = 1, 2, 3,…, 284 represents China’s 284 prefecture-level and above cities, while t = 2008, 2012,…, 2021 represents the year variable. Control includes control variables, $\varepsilon_{\mathrm{it}}$ is the random disturbance term, $\mu_{t}$ accounts for the time fixed effects, and $\delta_{i}$ captures city-specific fixed effects. Coefficients α , β and γ represent the respective coefficients.

### 4.2 Variable selection

(1) Urban Economic Resilience ( $res_{\mathrm{it}}$ ). Urban economic resilience is defined in this paper as a city’s ability to resist, recover, renew, and reconstruct in response to external uncertainties. The strength of this resilience can be measured using the sensitivity index method. With the actual GDP growth rate as the core indicator, this approach not only dynamically reflects the characteristics of urban economic fluctuations but also effectively controls for the influence of national economic cycle variations, ensuring the objectivity and comparability of the measurement. Furthermore, the sensitivity index method quantifies the strength of urban economic resilience while maintaining the continuity and consistency of the indicator, facilitating both long-term vertical tracking and horizontal comparisons across regions. The calculation formula (Equation 4) is as follows:

(4) $${\mathrm{resi}}_{\mathrm{it}}=\frac{\Delta GDP_{r,t}/GDP_{r,t-1}}{\Delta GDP_{n,t}/G_{n,t-1}}$$

Here, $res_{\mathrm{it}}$ represents urban economic resilience, $\Delta GDP_{r,t}/GDP_{r,t-1}$ denotes the GDP growth rate of city i in year t, and $\Delta GDP_{n,t}/GDP_{n,t-1}$ indicates the national GDP growth rate in year t. To ensure comparability, the continuity of the sensitivity index is maintained in this study.

(2) Explanatory Variable: Public Health Expenditure ( $pub_{\mathrm{it}}$ ). Per capita urban health expenditure offers a strong theoretical foundation and statistical advantages. It accurately reflects individual-level benefits, enhances the reliability of cross-city comparisons, and mitigates the influence of population size and economic level. This provides a more robust framework for analyzing the relationship between public health expenditure and urban economic resilience. In this study, per capita urban health expenditure is used as a proxy for public health expenditure.
(3) Mediating Variables. Technological Innovation ( $\ln\mathrm{inn}o_{\mathrm{it}}$ ): Technological innovation plays a pivotal mediating role in the relationship between public health expenditure and urban economic resilience. By advancing the modernization of public health systems, fostering the development of the health sector, accelerating economic structural upgrades, and enhancing social stability and resource allocation efficiency, technological innovation strengthens the impact of public health spending, thereby bolstering urban economic resilience. This study analyzes the number of patent applications in each city to assess the level of technological innovation and its regional disparities, illustrating how public health expenditure fosters urban economic resilience through technological advancement. Using patent applications as a measure of technological progress is both scientifically sound and practical. Patent data directly reflects the output of technological innovation and is closely linked to public health expenditure in areas such as research and development investment and technological innovation. In this study, the number of patent applications per 10,000 people is measured using the logarithmic value.

*Per Capita* GDP ( $\ln\mathrm{pergd}p_{\mathrm{it}}$ ): Per capita GDP serves as a key mechanism through which public health expenditure fosters urban economic resilience. It effectively captures the impact of public health spending on multiple dimensions, including economic growth, the labor market, economic structural adjustments, and social equity and stability. By increasing public health expenditure, labor productivity is directly enhanced, leading to higher per capita GDP, which in turn improves public health, optimizes industrial structure, promotes social welfare and equity, and ultimately strengthens urban economic resilience. In this study, the natural logarithm of per capita GDP is used as the measurement.

(4) Control Variables. The study includes the following control variables to account for additional factors influencing urban economic resilience: Industrial Structure: Measured as the ratio of value-added from the tertiary sector to value-added from the secondary sector. Openness to Trade: Measured as the ratio of total imports and exports (converted to RMB using the annual exchange rate) to GDP. Urban Land Use: Measured as the logarithm of the urban built-up area. Population Size: Measured as the logarithm of the urban permanent population. Urban Resident Income: Measured as the logarithm of the average income of urban residents. Scientific Expenditure: Measured as the ratio of scientific research expenditure to GDP.

### 4.3 Data sources

The 2008 global financial crisis prompted China to implement an economic stimulus plan, alongside significant increases in public health investment. Concurrently, the new healthcare reform plan was introduced, with the government expanding public health expenditure, thereby reflecting the long-term effects of this policy shift on urban economic resilience. Based on this context, the data used in this study consists of panel data from 284 cities at or above the prefecture level, spanning from 2008 to 2021. The data sources include the *China Regional Economic Statistical Yearbook*, *China Urban Statistical Yearbook*, and statistical bulletins from various provinces and cities. Missing data were addressed using linear interpolation. Descriptive statistics for the main variables are provided in Table 1.

**Table 1:** Descriptive statistical analysis.

Variable name	Abbreviation	Definition	Mean	Std. Dev.	Min	Max
Urban economic resilience	res	Measured using the sensitivity index method	1.430	0.284	0.103	3.018
Public health expenditure	pub	Per capita urban health expenditure	0.746	0.468	0.001	5.285
Industrial structure level	adv	Ratio of value added by the tertiary sector to the secondary sector	6.495	0.355	5.517	7.656
Openness to trade	tra	Ratio of total imports and exports to GDP	0.183	0.305	0.000	3.278
Urban construction land	bui	Logarithm of urban built-up area	4.506	0.862	1.945	7.405
Population size	pop	Logarithm of permanent population	5.866	0.705	3.043	8.074
Urban resident income	town	Logarithm of urban residents’ income	2.221	0.772	0.675	5.923
Scientific expenditure	rd	Ratio of scientific expenditure to GDP	0.259	0.262	0.128	6.309

## 5 Estimation and result

### 5.1 Benchmark regression analysis

Before empirically examining the relationship between public health expenditure and urban economic resilience, it is essential to address potential collinearity and data stationarity issues among the dependent variable, key explanatory variable, and control variables to avoid spurious regression results. A Variance Inflation Factor (VIF) test was conducted, showing a mean VIF of 2.19 and a maximum value of 3.28, both well below the critical threshold of 10. These results confirm the absence of multicollinearity among the variables, allowing for their inclusion in the regression model. Furthermore, a Hausman test comparing two-way fixed effects and two-way random effects models rejects the null hypothesis at the 1% significance level, validating the appropriateness of using a two-way fixed effects model for the analysis.

The two-way fixed effects model effectively controls for both time and individual fixed effects, thereby improving the accuracy of estimates concerning the impact of public health expenditure on urban economic resilience. This approach mitigates omitted variable bias, enhancing both the reliability and robustness of the results. The regression results using the two-way fixed effects model are presented in Table 2. Column (1) reports the results without control variables, where the coefficient for public health expenditure is 0.105 and significant at the 1% level, indicating that public health expenditure has a positive and significant effect on urban economic resilience. Columns (2) to (7) progressively introduce control variables, including industrial structure, foreign trade, urban construction land, population size, urban residents’ income, and scientific expenditure, using a stepwise regression approach. Across all models, the coefficients for public health expenditure remain significant at the 1% level, demonstrating its robust and stable positive impact on urban economic resilience. This finding suggests that public health expenditure directly strengthens a city’s ability to manage public health crises, reducing the adverse economic effects of such crises, ensuring the continuity of economic activities, and enhancing the city’s capacity to withstand shocks. Consequently, public health expenditure plays a crucial role in supporting the long-term stability and development of urban economies.

**Table 2:** Basic regression results.

Variables	(1)	(2)	(3)	(4)	(5)	(6)	(7)
pub	0.105***	0.110***	0.107***	0.093***	0.121***	0.118***	0.116***
	(0.013)	(0.013)	(0.013)	(0.012)	(0.012)	(0.012)	(0.012)
adv		0.223***	0.240***	0.182***	0.227***	0.224***	0.216***
		(0.031)	(0.031)	(0.030)	(0.030)	(0.030)	(0.030)
tra			−0.100***	−0.131***	−0.075***	−0.044*	−0.034
			(0.024)	(0.024)	(0.023)	(0.023)	(0.023)
bui				0.329***	0.296***	0.296***	0.293***
				(0.019)	(0.019)	(0.019)	(0.019)
pop					0.452***	0.350***	0.316***
					(0.035)	(0.036)	(0.036)
town						0.141***	0.137***
						(0.015)	(0.015)
rd							0.081***
							(0.015)
Constant	1.353***	−0.056	−0.140	−1.137***	−3.942***	−3.527***	−3.270***
	(0.010)	(0.197)	(0.198)	(0.199)	(0.289)	(0.289)	(0.292)
Observations	3,976	3,976	3,976	3,976	3,976	3,976	3,976
R-squared	0.491	0.504	0.508	0.575	0.612	0.630	0.636
Individual effect	YES	YES	YES	YES	YES	YES	YES
Time effect	YES	YES	YES	YES	YES	YES	YES

Standard errors in parentheses (***p < 0.01, **p < 0.05, *p < 0.1).

The regression results indicate that several control variables—industrial structure, urban construction land, population size, urban residents’ income, and scientific expenditure—positively and significantly contribute to urban economic resilience, while foreign trade openness has a negative impact: A diversified industrial structure reduces reliance on a single industry, enabling cities to spread risks during economic crises or industrial shocks. This diversification enhances economic resilience and stability. Optimizing and rationally allocating urban construction land improves urban environments, unlocks growth potential, and strengthens the city’s capacity for sustainable economic development. High population size fosters greater consumer demand, creating larger and more diverse markets. This sustained demand provides consistent momentum for economic recovery and growth. Higher incomes not only boost consumption but also strengthen savings capacity. Under balanced market conditions, increased savings can translate into government expenditure and corporate investments, supporting infrastructure development and economic resilience. Greater investment in research and development drives technological advancements, fosters innovation, and diversifies economic systems. This provides a strong foundation for economic recovery and sustained growth, enhancing resilience against future shocks. Conversely, the coefficient for foreign trade openness is negative, indicating that higher levels of trade dependence increase a city’s exposure to external market fluctuations. During periods of global economic instability, external shocks are more likely to propagate through trade, capital flows, and supply chains, intensifying domestic economic volatility and undermining resilience.

### 5.2 Robustness tests

#### 5.2.1 Excluding municipalities

Due to their unique administrative status, industrial concentration, and resource advantages, municipalities often exhibit significantly different levels of economic development, policy support, and public health expenditure compared to other regions. These differences may introduce bias into the overall regression results. To address this, the municipalities were excluded from the sample to better reflect the actual development dynamics of ordinary regions and to provide a clearer understanding of the relationship between public health expenditure and urban economic resilience. Column (1) of Table 3 presents the results after excluding the four municipalities. The coefficient for public health expenditure remains significantly positive at the 1% level, confirming the robustness of the baseline regression model.

**Table 3:** Robustness test.

Variables	Excluding municipalities	Changing dependent variable	Changing estimation method	Endogeneity test	Adjusting sample period
	(1)	(2)	(3)	(4)	(5)
pub	0.117***	0.060***	0.084***	0.123***	0.214***
	(0.012)	(0.015)	(0.012)	(0.016)	(0.019)
Control variables	YES	YES	YES	YES	YES
Individual effect	YES	YES	YES	YES	YES
Time effect	YES	YES	YES	YES	YES

Standard errors in parentheses (***p < 0.01, **p < 0.05, *p < 0.1).

#### 5.2.2 Changing the dependent variable

The findings thus far indicate that public health expenditure contributes to the enhancement of urban economic resilience. However, it remains necessary to determine whether this effect stems from the intrinsic role of public health or whether the integration of urban economic resilience in the economic domain influences the baseline regression results. To verify the reliability and generalizability of the model and ensure that the conclusions are not biased or overly dependent on the choice of the dependent variable, the robustness test is conducted by modifying the dependent variable. In this test, urban economic resilience is considered as a dynamic adjustment process. Drawing on the concept of the deviation share method, urban resilience is calculated by assessing the deviation of each city’s annual actual GDP from its actual GDP in 2008. The formula (Equation 5) used to calculate urban economic resilience for the 2008–2021 period is as follows:


(5) $$resi_{\mathrm{it}}=\frac{GDP_{\mathrm{it}}-GDP_{i2008}}{GDP_{i2008}}$$

Where $GDP_{\mathrm{it}}$ is the actual GDP of city i in year t, $GDP_{i2008}$ is the actual GDP of city i in 2008, and $resi_{\mathrm{it}}$ represents the urban economic resilience of city i during period t. A higher value for this indicator suggests that a city possesses stronger stability and corrective capacity, enabling it to navigate complex and dynamic internal and external environments more effectively. Such cities are better equipped to mitigate short-term shocks, restore economic order swiftly, optimize growth trajectories, reallocate resources, and achieve rapid economic recovery. Column (2) of Table 3 presents the results of this robustness test using the modified dependent variable. The regression results indicate that the coefficient for public health expenditure remains significantly positive at the 1% level, with the same direction as observed in the baseline regression. This further confirms the robustness of the baseline model and reinforces the reliability and scientific validity of the relationships between the variables.

#### 5.2.3 Revising the estimation approach

The Tobit model, when applied within a fixed-effects framework for panel data analysis, leverages cross-sectional variations between individuals as well as temporal dynamics. This dual capacity allows the model to maximize the use of panel data, enhancing the efficiency of parameter estimation. It is particularly suited for contexts where truncated data and individual-specific effects coexist. Given that the quantified measures of urban economic resilience in this study are all non-negative, a fixed-effects panel Tobit model is adopted to more precisely examine the relationship between public health expenditure and urban economic resilience.

The model (Equation 6) can be expressed as follows:


(6) $${{res}^{*}}_{\mathrm{it}}=\beta_{0}+\beta_{1}{\mathrm{pub}}_{\mathrm{it}}+\beta_{2}X_{\mathrm{it}}+\xi_{i}+\varepsilon_{\mathrm{it}}$$

where $re{s^{*}}_{\mathrm{it}}$ represents the latent (unobserved) urban economic resilience for city i at time t; $pub_{\mathrm{it}}$ denotes the core explanatory variable, public health expenditure; $\beta_{1}$ is the coefficient of interest; $\xi_{i}$ captures individual fixed effects, accounting for time-invariant heterogeneity across cities; and $\varepsilon_{\mathrm{it}}$ is the error term.

The fixed-effects panel Tobit model accommodates the non-standard distribution resulting from the interplay of fixed effects and data truncation. Traditional Ordinary Least Squares (OLS) estimation methods are therefore inadequate, and conditional maximum likelihood estimation (MLE) is instead employed. As shown in column (3) of Table 3, the regression results align closely with those from the baseline model, demonstrating that the baseline results are not overly dependent on specific model specifications. This reinforces the generalizability and robustness of the conclusions.

#### 5.2.4 Addressing endogeneity

While public health expenditure likely exerts a positive effect on urban economic resilience, it is equally plausible that regions with higher resilience could influence public health spending, introducing potential reverse causality. This bidirectional relationship could result in endogeneity, wherein public health expenditure is correlated with the error term, compromising the consistency and unbiasedness of the regression estimates. To address this issue, a partial linear instrumental variable model (Equations 7, 8) based on double machine learning is constructed:


(7) $$res_{\mathrm{it}}=\mathrm{\theta pub}+g\left(X_{\mathrm{it}}\right)+\varepsilon_{\mathrm{it}}$$

(8) $$IV_{\mathrm{it}}=m\left(X_{\mathrm{it}}\right)+V_{\mathrm{it}}$$

where $IV_{\mathrm{it}}$ serves as the instrumental variable for $pub_{\mathrm{it}}$ . In this study, we use the average public health expenditure of other cities within the same province during the same year as the instrumental variable. This measure reflects inter-city interactions in public health spending within a region, satisfying the relevance criterion for instrumental variables. Furthermore, historical data on public health expenditures from the previous year at the provincial level is used, as it is unlikely to be directly related to the current level of urban economic resilience, satisfying the exogeneity assumption. Using this methodology, an endogeneity test was performed, and the results are presented in column (4) of Table 3. The regression coefficient for public health expenditure is 0.123, statistically significant at the 1% level. These findings confirm that public health expenditure positively contributes to urban economic resilience, supporting the consistency and reliability of the baseline regression results.

#### 5.2.5 Adjusting the study period

The outbreak of COVID-19 in December 2019 triggered a sharp increase in public health expenditures. These abnormal changes in expenditure during the pandemic may render the regression results overly sensitive to data from these exceptional years, thereby affecting the model’s robustness. Additionally, the pandemic posed unprecedented challenges to urban economic resilience, with varying capacities among cities to respond. This may have altered the relationship between the variables and increased the instability of the regression results. To address this concern, the sample period was restricted to 2010–2019, excluding the years influenced by the pandemic. The regression analysis was then re-conducted, with results shown in column (5) of Table 3. The coefficient for public health expenditure is 0.214, statistically significant at the 1% level. These findings remain consistent with those from the baseline regression, confirming the robustness of the results when accounting for potential temporal effects.

### 5.3 Mechanism analysis

The preceding analysis established the positive effect of public health expenditure on urban economic resilience. The following section explores the underlying mechanisms driving this relationship. Specifically, we investigate whether technological innovation and per capita GDP serve as channels through which public health expenditure influences urban economic resilience. To this end, a stepwise regression approach is adopted. The regression results based on Models 2 and 3 are presented in Table 4.

**Table 4:** Results of mechanism analysis.

	(1)	(2)	(3)	(4)
Variables	lninno	res	lnpergdp	res
pub	0.145***	0.108***	0.032***	0.091***
	(0.038)	(0.012)	(0.008)	(0.010)
lninno		0.053***		
		(0.005)		
lnpergdp				0.774***
				(0.022)
Control variables	YES	YES	YES	YES
Individual effect	YES	YES	YES	YES
Time effect	YES	YES	YES	YES
Constant	−5.754***	−7.278***	−1.391^***^	−2.968^***^
	(0.368)	(1.155)	(0.068)	(0.115)
Observations	3,976	3,976	3,976	3,976
R-squared	0.948	0.759	0.981	0.813
Indirect effect	0.008		0.065	
Direct effect	0.108		0.091	
Proportion of indirect effect	6.90%		21.55%	

Standard errors in parentheses (***p < 0.01, **p < 0.05, *p < 0.1).

Column (1) of Table 4 shows that public health expenditure significantly enhances urban technological innovation. This increase drives the integration of disciplines such as medicine, information technology, and big data, addressing the specific needs of the public health sector for precise monitoring, disease diagnosis, and efficient prevention strategies. Moreover, it fosters interdisciplinary collaboration and accelerates the application of innovative technologies, leading to breakthroughs in emerging cross-disciplinary fields. Column (2) incorporates technological innovation into the regression model, where the mediating variable remains statistically significant at the 1% level. The Sobel test produces a Z-statistic of 3.59, indicating strong statistical significance, with the mediating effect accounting for 6.90% of the total effect. These findings suggest that public health expenditure promotes technological innovation, thereby enhancing cities’ ability to resist external shocks. This study further highlights that sustained investment in public health accelerates the development of advanced technologies, including telemedicine, diagnostic tools for public health emergencies, and health-focused big data solutions. These innovations optimize medical resource allocation, improve the efficiency and accuracy of healthcare services, significantly shorten emergency response times, and reduce healthcare costs. Collectively, these advancements enhance urban resilience, optimize structural configurations, and help cities transition to a new equilibrium in economic resilience.

Column (3) of Table 4 indicates that public health expenditure (pub) has a significant and positive effect on per capita GDP. This result suggests that public health investment improves the overall health of residents, reduces the incidence of disease, and alleviates the financial burden of healthcare. These improvements enable a more productive workforce, boosting labor efficiency and driving per capita GDP growth. Column (4) demonstrates that both public health expenditure and per capita GDP have statistically significant positive effects at the 1% level. This finding indicates that public health expenditure indirectly enhances urban economic resilience by increasing per capita GDP. The Sobel test confirms this relationship, with a Z-statistic of 3.97 and the mediating effect of per capita GDP accounting for 21.55% of the total effect. This substantial mediating role underscores the importance of per capita GDP as a pathway through which public health expenditure enhances urban economic resilience. The analysis further reveals that robust public health services reduce the prevalence of chronic illnesses, occupational diseases, and infectious diseases, enabling workers to participate in economic activities with greater energy and efficiency. This “health dividend” increases individual economic contributions and enhances collective labor productivity, ultimately strengthening the economic competitiveness of cities. Over the long term, the health dividend not only improves individual productivity but also enhances overall urban resilience by boosting collective economic performance.

### 5.4 Heterogeneity analysis

Using the economic regional divisions defined by the National Bureau of Statistics, the sample cities are grouped into three categories: 100 cities in the eastern region, 96 cities in the central region, and 84 cities in the western region. The regression results are presented in Columns (1), (2), and (3) of Table 5. For cities in the eastern region, the coefficient of public health expenditure is significantly positive at the 1% level, with the largest magnitude of 0.361. This indicates that public health expenditure has a clear and substantial promoting effect in this region. For cities in the central region, the coefficient is also significantly positive at the 1% level, with a moderate magnitude of 0.42, demonstrating a similar but slightly less pronounced effect. In contrast, the coefficient for cities in the western region is −0.029, non-significant, and the smallest among the three regions, suggesting that public health expenditure has a weak and non-significant suppressing effect on economic resilience in this area. These results reveal regional disparities in the response to public health expenditure, reflecting the uneven nature of regional economic development. In the eastern region, where economic development is more advanced and infrastructure is more robust, public health expenditure achieves higher marginal returns. This expenditure is more effectively converted into productivity gains and social stability, thereby exerting a significant positive impact on urban economic resilience. In the central region, where economic development is intermediate, public health expenditure helps address developmental gaps and improves public health outcomes. However, due to a relatively weaker economic base and less efficient resource allocation, the effect is somewhat lower than in the eastern region. Meanwhile, in the western region, where public health infrastructure is underdeveloped, new investments are often directed toward basic infrastructure construction. Such investments may take longer to yield economic benefits and may even temporarily suppress economic resilience due to the scale and time required for implementation.

**Table 5:** Heterogeneity analysis.

	(1)	(2)	(3)	(4)	(5)	(6)	(7)
Variables	Eastern cities	Central cities	Western cities	Provincial capital cities	Non-provincial capital cities	Non-resource-based cities	Resource-based cities
pub	0.361***	0.042***	−0.029	0.102***	0.121***	0.109***	0.127***
	(0.028)	(0.016)	(0.026)	(0.030)	(0.013)	(0.016)	(0.019)
Control variables	YES	YES	YES	YES	YES	YES	YES
Individual effect	YES	YES	YES	YES	YES	YES	YES
Time effect	YES	YES	YES	YES	YES	YES	YES
Constant	−3.250***	−3.402***	−0.536	−2.290**	−3.235***	−2.229***	−4.300***
	(0.589)	(0.427)	(0.525)	(0.974)	(0.319)	(0.351)	(0.523)
N	100	100	84	30	254	170	114
R-squared	0.652	0.572	0.573	0.578	0.641	0.596	0.610

Standard errors in parentheses (***p < 0.01, **p < 0.05, *p < 0.1).

To explore the differing effects of public health expenditure on economic resilience in provincial capital cities versus non-capital cities, the sample is divided into 30 provincial capital cities and 254 non-capital cities for regression analysis. The results are shown in Columns (4) and (5) of Table 5. For provincial capital cities, the coefficient for public health expenditure is 0.102, which is statistically significant at the 1% level, indicating that public health expenditure has a positive impact on economic resilience in these cities. For non-capital cities, the coefficient is 0.121, also statistically significant at the 1% level, showing that public health expenditure similarly promotes economic resilience. Notably, the coefficient for non-capital cities is larger than that for provincial capital cities, suggesting that public health expenditure has a more substantial impact on the economic resilience of non-capital cities.

The findings of the study reveal that public health expenditure has a more significant effect on improving the economic resilience of non-provincial capitals compared to provincial capitals. This is because non-provincial capitals typically have weaker public health infrastructure and resource allocation, making their economic resilience more vulnerable. In contrast, provincial capitals already possess stronger economic resilience, making further improvements more challenging. As a result, non-provincial capitals have greater potential for enhancing their economic resilience, which explains why public health expenditure has a more substantial impact in these cities.

Following the National Plan for Sustainable Development of Resource-Based Cities (2013–2020) and the 14th Five-Year Plan for Promoting High-Quality Development in Resource-Based Areas, the 284 cities in the sample are categorized into 170 non-resource-based cities and 114 resource-based cities. The regression results are presented in Columns (6) and (7) of Table 5. The results show that public health expenditure has a statistically significant positive effect on economic resilience at the 1% level in both types of cities. Specifically, the coefficient for non-resource-based cities is 0.109, while for resource-based cities, it is 0.127. These findings suggest that the positive impact of public health expenditure on economic resilience is more pronounced in resource-based cities. The study finds that resource-based cities, confronted with challenges such as resource depletion and environmental pollution, must urgently undergo industrial transformation to strengthen their sustainability. Increasing public health expenditure can improve the living conditions of urban residents and enhance the economic resilience of cities.

## 6 Further analysis based on spatial effects

As the economy transitions toward high-quality development, the free flow of labor, capital, and technology has significantly enhanced intercity collaboration and interaction, strengthening the spatial interconnections of urban economic resilience. Cities with high levels of public health expenditure often act as exemplars, guiding neighboring cities to improve resource allocation efficiency. This demonstration effect expands through imitation, amplifying the overall impact. From a spatial perspective, this study systematically examines the spatial mechanisms between public health expenditure and urban economic resilience, focusing on whether public health expenditure enhances economic resilience through spatial spillover effects.

### 6.1 Spatial autocorrelation test

Spatial autocorrelation is a prerequisite for spatial regression models. Testing for spatial autocorrelation ensures the inclusion of spatial factors, avoiding biases caused by traditional models that overlook spatial dependencies. This test also reveals whether variables influence adjacent regions, i.e., whether spatial spillover effects exist. In this study, Moran’s I index is constructed using a spatial adjacency matrix and a spatial economic-geographic nested matrix to test for spatial effects between public health expenditure and urban economic resilience. Table 6 reports the Moran’s I indices for urban economic resilience from 2008 to 2021 under both matrices. The results indicate that urban economic resilience passes the significance test at the 1% level, with the mean Moran’s I index greater than 0. This confirms the existence of significant positive spatial relationships. The study reveals that the underlying logic of economic networks between cities highlights the crucial role of enhancing the economic resilience of core cities in strengthening the risk resistance of surrounding cities and the broader region, particularly within the context of regional integration and coordinated development.

**Table 6:** Moran’s I index for urban economic resilience.

Year	Adjacency matrix	Economic-geographic nested matrix	Year	Adjacency matrix	Economic-geographic nested matrix
	Moran’s I	Moran’s I		Moran’s I	Moran’s I
2008	0.385***	0.087***	2015	0.346***	0.100***
	(9.806)	(2.744)		(8.773)	(3.168)
2009	0.437***	0.135***	2016	0.402***	0.104***
	(11.116)	(4.265)		(10.180)	(3.300)
2010	0.426***	0.157***	2017	0.458***	0.104***
	(10.864)	(4.939)		(11.592)	(3.307)
2011	0.420***	0.173***	2018	0.499***	0.115***
	(10647)	(5.417)		(12.609)	(3.643)
2012	0.406***	0.166***	2019	0.535***	0.121***
	(10.296)	(5.199)		(13.516)	(3.827)
2013	0.344***	0.141***	2020	0.529***	0.111***
	(8.730)	(4.433)		(13.371)	(3.513)
2014	0.333***	0.111***	2021	0.533***	0.116***
	(8.441)	(3.510)		(13.476)	(3.665)

The values in parentheses represent Z-scores. Standard errors in parentheses (***p < 0.01, **p < 0.05, *p < 0.1).

### 6.2 Spatial econometric model design

To evaluate whether public health expenditure exhibits spatial spillover effects on urban economic resilience, this study employs the OLE-SAR and SEM-SDM modeling frameworks, establishing the following regression models (Equations 9, 10):


(9) $$\begin{array}{l} res_{\mathrm{it}}=\mu_{i}+\gamma_{t}+\mathrm{\rho Wre}s_{\mathrm{it}}+\beta_{1}pub_{\mathrm{it}}+\beta_{2}X_{\mathrm{it}}\\ +\theta_{1}Wpub_{\mathrm{it}}+\theta_{1}WX_{\mathrm{it}}+\omega_{\mathrm{it}} \end{array}$$

(10) $$\omega_{\mathrm{it}}=\lambda W\omega_{\mathrm{it}}+\varepsilon_{\mathrm{it}}$$

λ=0

ρ=0

θ=0

Where: i and t represent cities and years, respectively. $res_{\mathrm{it}}$ denotes urban economic resilience. ${\mathrm{pub}}_{\mathrm{it}}$ represents public health expenditure. $X_{\mathrm{it}}$ captures relevant control variables. $\mu_{i}$ and $\gamma_{t}$ indicate individual and time effects, respectively. ρ the spatial autoregressive coefficient. λ represents the spatial error term coefficient. *θ* denotes the coefficient of spatial interaction terms. W is the spatial weight matrix. *β* is the regression coefficient vector. $\omega_{\mathrm{it}}$ and $\varepsilon_{\mathrm{it}}$ are random disturbance terms.

### 6.3 Analysis of spatial effects

The regression results presented in Table 7, derived from the Spatial Durbin Model (SDM) using both the spatial adjacency matrix and the economic-geographic nested matrix, yield the following key insights: The coefficients of public health expenditure are positive and statistically significant at the 1% level under both matrices. This demonstrates that public health spending has a direct and significant positive impact on the economic resilience of cities. Notably, the effect is more pronounced under the economic-geographic nested matrix, suggesting that broader economic linkages amplify the benefits of public health investment. The spatial lag term coefficients are also significantly positive under both matrices, indicating that public health expenditure generates positive spillover effects on the economic resilience of neighboring cities. The effect is stronger under the economic-geographic nested matrix, highlighting the role of economic connectivity in facilitating the transmission of benefits across cities. The spatial autocorrelation coefficients are significantly positive in both models, confirming the presence of spatial interdependence in urban economic resilience. Under the adjacency matrix, the spatial dependency is stronger, underscoring the influence of geographical proximity in shaping intercity economic dynamics. Research findings, the positive and significant coefficients for public health expenditure reinforce its critical role in enhancing urban economic resilience. The stronger effects observed under the economic-geographic nested matrix indicate that cities with greater economic integration are better positioned to leverage public health spending, enabling faster recovery and greater risk mitigation. The significant spatial lag term coefficients suggest that public health spending not only benefits the local city but also positively influences the economic resilience of neighboring cities. This spillover effect likely stems from regional cooperation, such as shared healthcare resources and institutional collaborations, which strengthen intercity resilience. The significantly positive spatial autocorrelation coefficients reveal a strong spatial dependency, meaning that a city’s economic resilience is influenced by the resilience levels of its surrounding cities. This highlights the interconnected nature of urban economic systems, where neighboring cities often share risks and resources. The study demonstrates that higher public health expenditure enhances a city’s resilience to risks and its economic recovery capacity, enabling faster recovery in the face of public health crises or other economic shocks. Furthermore, it promotes mutual benefits in neighboring areas through shared health resources and regional healthcare cooperation. In this way, the benefits of public health spending extend beyond the local city, strengthening the economic resilience of the entire region through collaboration and interaction.

**Table 7:** Regression results for the spatial Durbin model.

Variables	(1)	(2)
	Adjacency matrix	Economic-geographic nested matrix
pub	0.041***	0.079***
	(0.009)	(0.010)
W × pub	0.028*	0.207*
	(0.015)	(0.030)
Control variables	YES	YES
Individual fixed effects	YES	YES
Time fixed effects	YES	YES
rho	0.553***	0.407***
	(0.014)	(0.025)
a2_e	0.012***	0.017***
	(0.000)	(0.000)
Log-likelihood	2983.005	2983.005
R-squared	0.501	0.501
Direct	0.049***	0.096***
	(0.010)	(0.011)
Indirect	0.099***	0.038***
	(0.024)	(0.045)
Total	0.148***	0.477***
	(0.028)	(0.049)
N	3,976	3,976

Standard errors in parentheses (***p < 0.01, **p < 0.05, *p < 0.1).

The relationship between public health expenditure (core variable) and urban economic resilience (dependent variable) is further analyzed through direct, indirect, and total effects: Direct effects capture the immediate impact of public health expenditure on a city’s economic resilience. Under the economic-geographic nested matrix, the direct effect is 0.096***, higher than the 0.049*** observed under the adjacency matrix. This reflects the advantages of economically interconnected regions, where public health investments are more effectively translated into local economic resilience through resource integration and technological diffusion. Indirect effects measure the spillover impact of a city’s public health expenditure on the economic resilience of neighboring cities. The indirect effect under the adjacency matrix is 0.099***, higher than the 0.038*** observed under the nested matrix. This suggests that geographical proximity plays a stronger role in facilitating short-distance diffusion of resources and technologies, reinforcing economic resilience in neighboring cities. Total effects combine the direct and indirect impacts of public health expenditure. The total effect under the economic-geographic nested matrix is 0.477***, significantly higher than the 0.148* observed under the adjacency matrix. This underscores the amplified impact of public health spending within economically integrated regional networks, where cross-city cooperation and shared economic activities strengthen overall resilience. Research findings, Public health expenditure has a multifaceted impact on urban and regional economic resilience, as reflected in its direct, indirect, and total effects: Public health spending improves local economic resilience by enhancing healthcare services, strengthening infrastructure, and increasing risk mitigation capabilities. The stronger direct effect under the economic-geographic nested matrix highlights the role of economic linkages in maximizing these benefits. The spillover effects of public health expenditure on neighboring cities are significant, primarily driven by mechanisms such as resource sharing, healthcare cooperation, and institutional learning. The adjacency matrix reveals that geographical proximity remains a critical factor in facilitating these short-distance spillover effects. The total effect of public health expenditure is significantly greater under the economic-geographic nested matrix, reflecting the stronger overall impact in regions with tighter economic connections. These networks enable cities to pool resources and collectively enhance their resilience, amplifying the benefits of public health spending across the region.

## 7 Research conclusions and practical implications

### 7.1 Research conclusions

This study utilizes panel data from 284 Chinese cities at the prefecture level and above, spanning 2008 to 2021, to investigate the relationship between public health expenditure and urban economic resilience. It explores the mechanisms of influence, heterogeneity, and spatial effects, offering both theoretical and empirical insights. The main conclusions are as follows: (1) Public health expenditure has a significant positive effect on urban economic resilience. It shortens economic recovery cycles and improves the ability of cities to withstand and recover from public health emergencies and other external shocks. (2) Public health expenditure indirectly strengthens urban economic resilience by driving technological innovation and increasing per capita GDP. Technological innovation improves the efficiency and quality of healthcare services, while higher per capita GDP contributes to resilience through health dividends and increased consumer spending, providing a stable foundation for urban economic development. (3) The impact of public health expenditure varies significantly across regions. Eastern cities benefit the most, with the strongest positive effects on economic resilience, followed by central cities, while the effect in western cities is weaker and, in some cases, even negative. These findings highlight regional imbalances in development and differences in resource allocation efficiency. Moreover, the marginal benefits of public health expenditure are more pronounced in non-capital cities and resource-based cities, reflecting the influence of economic structure and development stage on the efficiency of public health spending. (4) Public health expenditure not only directly enhances the economic resilience of local cities but also generates significant positive spillover effects on neighboring cities through mechanisms such as regional resource sharing and technology diffusion. These spillover effects are particularly pronounced in economically interconnected regions, as captured by the economic-geographic nested matrix.

### 7.2 Practical implications

Based on the research findings, the following policy recommendations are proposed: (1) Optimize the Structure and Allocation of Public Health Expenditure: Eastern regions should further enhance the innovation-driven role of public health spending. Central regions can achieve steady economic growth through the synergistic effects of public health investment and other infrastructure development. In western regions, there should be increased investment in infrastructure, improved efficiency in fund utilization, and a focus on promoting equitable healthcare coverage. This will help avoid the negative impact of excessive spending on economic resilience in the short term. (2) Develop Region-Specific Policies: For non-provincial capital cities, it is essential to optimize healthcare system development while increasing financial support to improve marginal returns. For resource-dependent cities, public health expenditure should be leveraged to facilitate economic transformation, increase the share of high-value-added services, reduce reliance on a single industry, and improve economic sustainability. (3) Establish Targeted Fiscal Subsidy Systems: Governments should implement special fiscal subsidies tailored to the public health needs and economic development conditions of different regions. In the western and underdeveloped regions, fiscal subsidies can accelerate the construction and upgrading of healthcare facilities, such as hospitals and clinics, thereby enhancing healthcare capacity and coverage. These subsidies should also support public health initiatives such as vaccination, disease prevention, and health education, ensuring effective implementation and broad outreach. Additionally, subsidies should attract highly skilled healthcare professionals to underdeveloped regions and support ongoing education and professional development for local medical staff, thereby improving overall healthcare service quality. (4) Optimize Investment and Financing Mechanisms for Public Health: Governments should streamline investment and financing mechanisms in the public health sector to enhance fund allocation efficiency and ensure balanced development across regions. First, public-private partnerships (PPPs) should be encouraged to attract private capital into public health projects, alleviating fiscal pressures and improving capital utilization. Second, dedicated public health funds should be established to support infrastructure development, technological innovation, vaccine production, and other key projects, ensuring sustained investment and growth. Third, tax incentives and other policies can be introduced to encourage private and social sector participation. Finally, careful allocation of fiscal funds, especially in western and underdeveloped areas, will help prevent wasteful spending. (5) Promote the Development of Digital Healthcare: The government should increase investment in digital health technologies, particularly in areas such as telemedicine, health big data, and artificial intelligence, to improve healthcare accessibility. By advancing telemedicine and smart health management applications, healthcare services can be delivered via the internet, addressing resource shortages and utilizing data analytics to enhance public health and reduce disease transmission. Additionally, a unified health information platform should be established to facilitate data sharing and integration, optimizing resource allocation and strengthening public health response capabilities. The government should also support the growth of digital health enterprises, foster technological innovation, and improve healthcare quality. Finally, investments in education and training programs for digital health professionals will help attract top talent, ensuring the continued development of the sector and improving healthcare service standards. (6) Strengthen Regional Collaboration and Resource Sharing: Governments should create a more integrated regional health cooperation framework to facilitate the flow of high-quality public health resources, reduce regional disparities, and leverage the technical and resource advantages of eastern and central regions. This will support the development of regional healthcare networks and enhance resource-sharing platforms. (7) Enhance International Cooperation and Experience Sharing: Strengthening technical cooperation and experience sharing with other countries in the field of public health is essential, especially in responding to major public health crises. By actively participating in global public health governance, countries can increase their influence and standing within the international health system.

## Data Availability

The original contributions presented in the study are included in the article/Supplementary material, further inquiries can be directed to the corresponding author.
